# Robotic-assisted repair of incisional hernia—early experiences of a university robotic hernia program and comparison with open and minimally invasive sublay technique (eMILOS)

**DOI:** 10.1007/s00423-023-03129-3

**Published:** 2023-10-12

**Authors:** Gabriel A. Plitzko, Björn-Ole Stüben, Anastasios Giannou, Matthias Reeh, Jakob R. Izbicki, Nathaniel Melling, Michael Tachezy

**Affiliations:** Department of General, Visceral and Thoracic Surgery, University Medical Center Hamburg-Eppendorf, Martini Str. 52, 20246 Hamburg, Germany

**Keywords:** Robotic hernia surgery, Incisional hernia, Robotic retro-muscular hernia repair, eTEP, eMILOS, Transverse abdominis release, Abdominal wall reconstruction

## Abstract

**Purpose:**

With robotic surgical devices, an innovative tool has stepped into the arena of minimally invasive hernia surgery. It combines the advantages of open (low recurrence rates and ability to perform complex procedure such as transverse abdominis release) and laparoscopic surgery (low rate of wound and mesh infections, less pain). However, a superiority to standard minimally invasive procedures has not yet been proven. We present our first experiences of robotic mesh repair of incisional hernias and a comparison of our results with open and minimally invasive sublay techniques.

**Methods:**

A retrospective analysis of all patients who underwent robotic-assisted mesh repair (RAHR) for incisional hernia between April and November 2022 (RAHR group) and patients who underwent open sublay (Sublay group) or eMILOS hernia repair (eMILOS group) between January 2018 and November 2022 was carried out. Patients in the RAHR group were matched 1:2 to patients in the Sublay group by propensity score matching. Patient demographics, preoperative hernia characteristics and cause of hernia, intraoperative variables, and postoperative outcomes were evaluated. Furthermore, a subgroup analysis of only midline hernia was performed.

**Results:**

A total of 21 patients received robotic-assisted incisional hernia repair. Procedures performed included robotic retro-muscular hernia repair (r-RMHR, 76%), with transverse abdominis release in 56% of the cases. In one patient, r-RHMR was combined with robotic inguinal hernia repair. Two patients (10%) were operated with total extraperitoneal technique (eTEP). Robotic-assisted transabdominal preperitoneal hernia repair (r-TAPP) was performed in three patients (14%).

Median (range) operating time in the RAHR group was significantly longer than in the sublay and eMILOS group (291 (122–311) vs. 109.5 (48–270) min vs. 123 (100–192) min, respectively, *p* < 0.001). The meshes applied in the RAHR group were significantly compared to the sublay (mean (SD) 529 ± 311 cm^2^ vs. 356 ± 231, *p* = 0.037), but without a difference compared to the eMILOS group (mean (SD) 596 ± 266 cm^2^). Median (range) length of hospital stay in the RAHR group was significantly shorter compared to the Sublay group (3 (2–7) vs. 5 (1–9) days, *p* = 0.032), but not significantly different to the eMILOS group. In short term follow-up, no hernia recurrence was observed in the RAHR and eMILOS group, with 9% in the Sublay group. The subgroup analysis of midline hernia revealed very similar results.

**Conclusion:**

Our data show a promising outcome after robotic-assisted incisional hernia repair, but no superiority compared to the eMILOS technique. However, RAHR is a promising technique especially for complex hernia in patients with relevant risk factors, especially immunosuppression. Longer follow-up times are needed to accurately assess recurrence rates, and large prospective trials are needed to show superiority of robotic compared to standard open and minimally invasive hernia repair.

## Introduction

Ventral hernias are among the most common conditions seen by surgeons around the world, with increasing prevalence in the West. In up to 28% of patients undergoing abdominal operations, incisional hernias occur in the postoperative course, resulting in estimated up to US$ 6.3 billion per year in healthcare costs in the US [1, 2].

While the advantages of mesh use are well documented, the optimal surgical technique and mesh placement are still the object of discussion and disagreement [3–7]. Comparisons of mesh placement regarding recurrence rates favor retro-muscular placement, with lower recurrence rates compared to inlay or onlay placement [4]. Despite overwhelming high-quality evidence, it is estimated that less than 50% of ventral hernias are repaired using mesh [8]. This may explain why treatment successes for ventral hernias are still moderate, with recurrence rates of 15–40% reported [9, 10].

Laparoscopic approaches were developed in recent years as an alternative to classic open hernia repair and are shown to have similar recurrence rates to open repair and are superior in terms of length of stay (LOS) and surgical site infections (SSI) [11]. Retro-muscular mesh placement using laparoscopic methods has been described, such as the eMILOS procedure (endoscopic mini/less open sublay technique) and the enhanced-view total extraperitoneal technique (eTEP) [12, 13].

Even though the laparoscopic method has proven advantages over open hernia repair, it has not been widely adopted by surgeons, with only 20% of ventral hernia repairs being performed laparoscopically [14]. This is presumably due to the technical difficulties and steep learning curve associated with the minimally invasive technique.

The newest development in the field is robotic-assisted hernia repair (RAHR). The 3D visualization along with wristed instruments and a more ergonomic seated position of the surgeon potentially make minimally invasive repair of ventral hernias more accessible. Furthermore, recent studies have shown a decrease in LOS and lower rates of complications for RAHR, especially in complex ventral hernia repair with the need for a transversus abdominis release (TAR) [15–17].

The aim of the current study was to present our early experiences, the operative techniques used, the patient characteristics, and the surgical outcomes in robotic incisional hernia surgery in comparison to the open and minimal-invasive sublay (eMILOS) technique.

## Methods

### Study population

All adult patients who underwent robotic-assisted retro-muscular or pre-peritoneal mesh repair for incisional hernia between April and November 2022 were included in this study and retrospectively reviewed (RAHR group). All operations were electively performed by one senior surgeon (M. T.). For the Sublay group and eMILOS group, all adult patients who had elective incisional hernia repair between January 2018 and November 2022 were included. Patients with recurrent hernia were excluded from the study. For the subgroup analysis of midline hernia, all lateral hernias were excluded.

Patient selection for the robotic-assisted technique was based on surgeon’s preference.

### Surgical technique

#### Robotic-assisted hernia repair

All procedures were performed using the da Vinci Xi surgical system (Intuitive Surgical Inc., Sunnyvale, CA, USA). Three different surgical techniques were used in this study: the robotic retro-muscular hernia repair (r-RMHR) technique with transversus abdominis release (TAR) if needed, the robotic-assisted transabdominal preperitoneal hernia repair (r-TAPP), and the enhanced-view totally extraperitoneal (eTEP) technique.

**Fig. 1:**
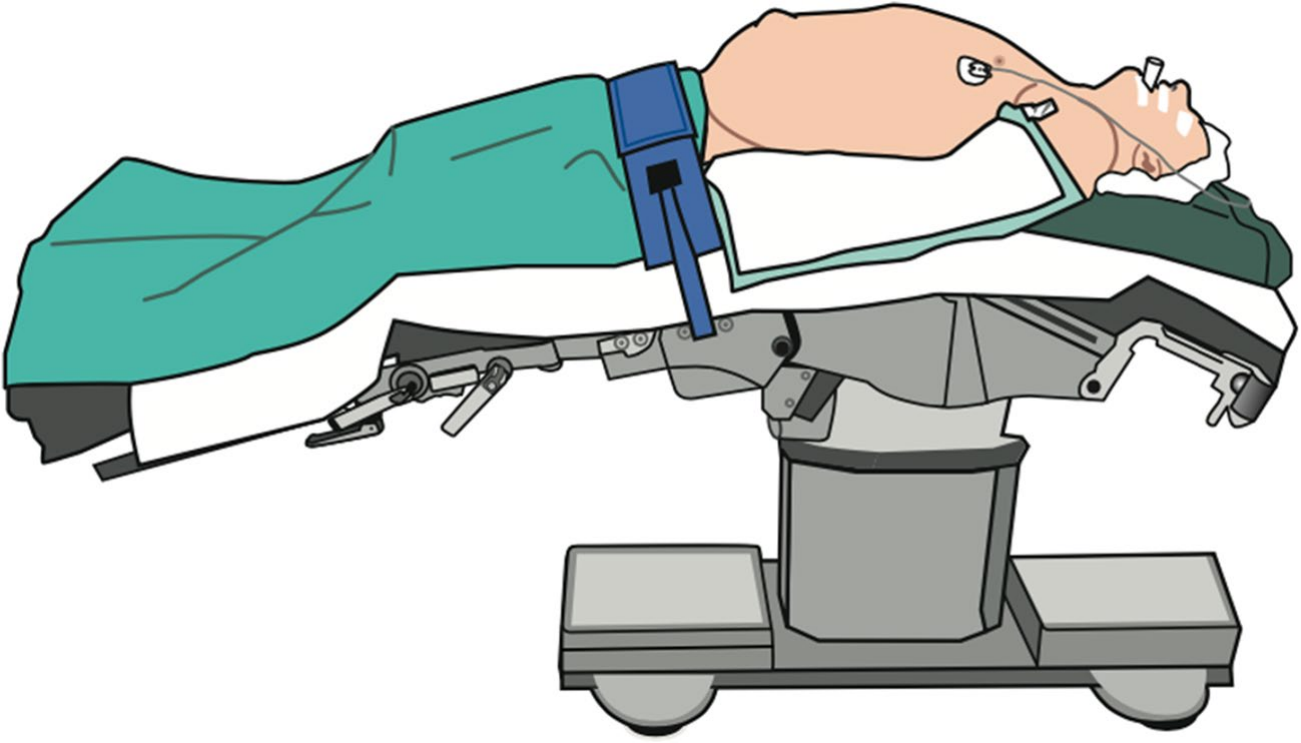
Patient positioning. Patient positioned supine on a flexed operating table. Both arms tucked alongside the body

**Fig. 2:**
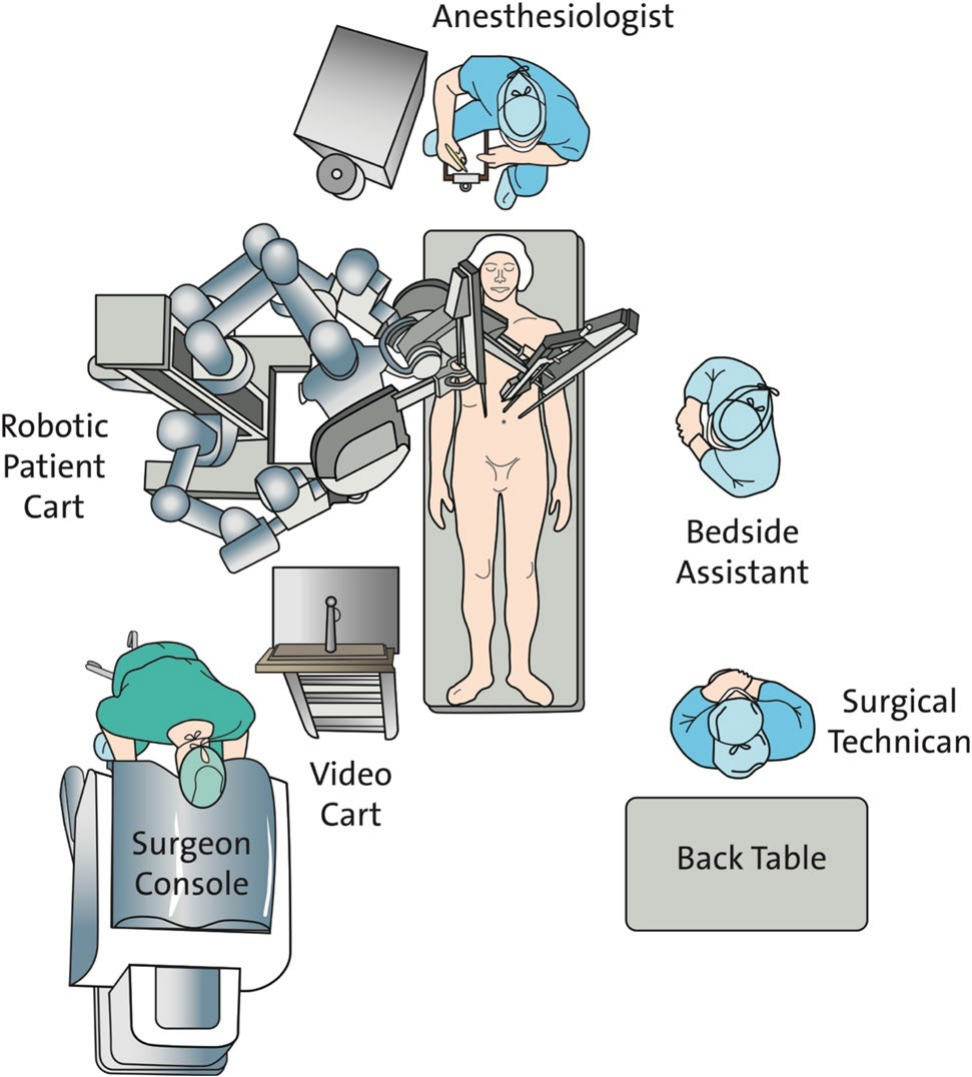
Operating room layout with lateral dock setup

For the eTEP and r-RMHR techniques, all patients were positioned supine with arms resting at the sides and tucked under a surgical drape. The operating table was flexed 15° to maximize the space between the costal margin and iliac crest and to prevent collision of the robotic arms (Fig. 1). The patient cart of the robot was placed on the right side of the patient (lateral dock setup), the bed-site assistant and the scrub nurse were positioned opposite to it (Fig. 2).

**Fig. 3:**
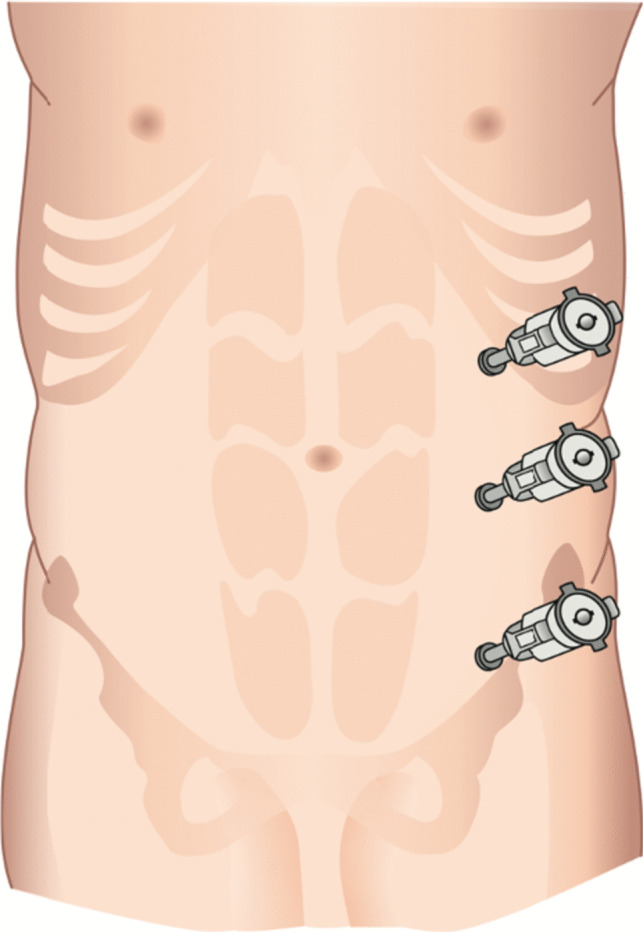
Trocar placement for r-RMHR-procedure

For r-RMHR procedure, the first 8-mm trocar (Intuitive Surgical Inc., Sunnyvale, CA, USA) was placed in subcostal position on the left side with a distance to the linea alba of about 12 cm. Additionally, two 8-mm trocars were placed on the same vertical line in the left flank under visual control. The distance between the trocars was at least 7 cm (Fig. 3). In the next step, hernia was reduced and adhesiolysis was performed if needed. To access the retromuscular plane, the ipsilateral posterior rectus sheath was opened slightly medial of the linea semilunaris. After complete mobilization of the ipsilateral posterior rectus fascia, the linea alba was crossed in the preperitoneal plane, and the contralateral rectus sheath was entered. Mobilization of the contralateral posterior rectus fascia was continued until the linea semilunaris was reached. The dissection was extended cranially and caudally to facilitate a mesh overlap of at least 5 cm to each site. The hernia defect was closed by a running suture, and the mesh was placed in the retromuscular plane. Closure of the ipsilateral posterior rectus fascia was accomplished with a running suture.

If needed, TAR was performed unilaterally at the contralateral side. In these cases, the posterior rectus sheath was opened from the xyphoid to the retropubic space, and its lateral border was exposed. Cranially, the fibers of the transversus abdominis were dissected at their insertion on the posterior rectus sheath. Then, the lateral detachments of the fascia of the transversus abdominis are released, and the dissection continued caudally into the retroinguinal/retropubis space and cranially across the costal margin onto the diaphragm.

**Fig. 4:**
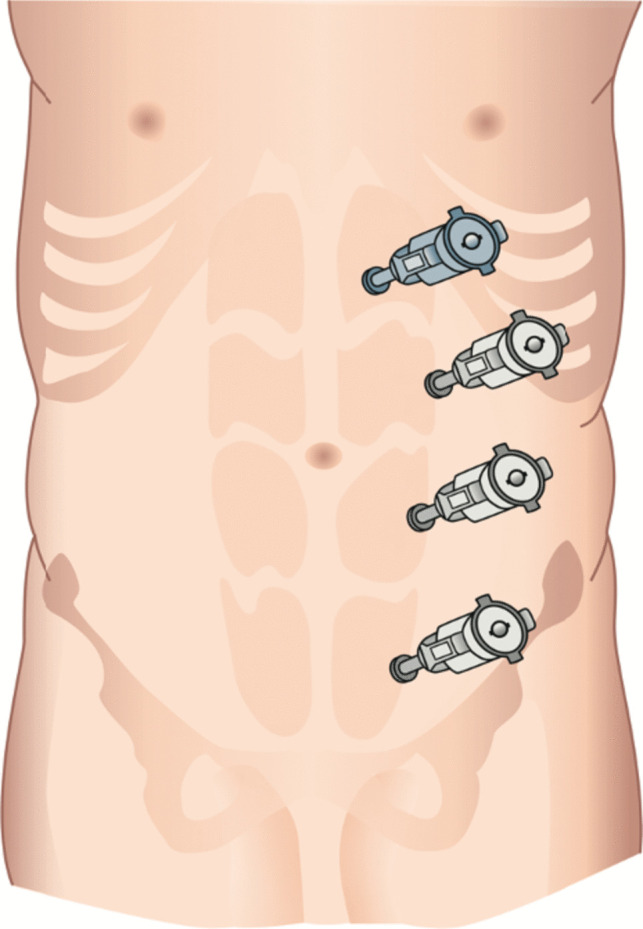
Trocar placement for eTEP-procedure (grey: 8-mm trocar, blueish: 12-mm optic trocar)

In eTEP procedure, the retro-rectus space was bluntly entered with a 12-mm optic trocar (Kii® Fios®, Applied Medical, Rancho Santa Margarita, CA, USA) in the epigastrium in the middle of the rectus sheet. The initial retro-rectus dissection was performed by blunt dissection with the camera. Subsequently, three additional 8-mm trocars were inserted under visualization at the lateral border of the rectus sheet (Fig. 4). The further steps of eTEP procedure were conducted in accordance to the description of Belyansky et al. [18].

**Fig. 5:**
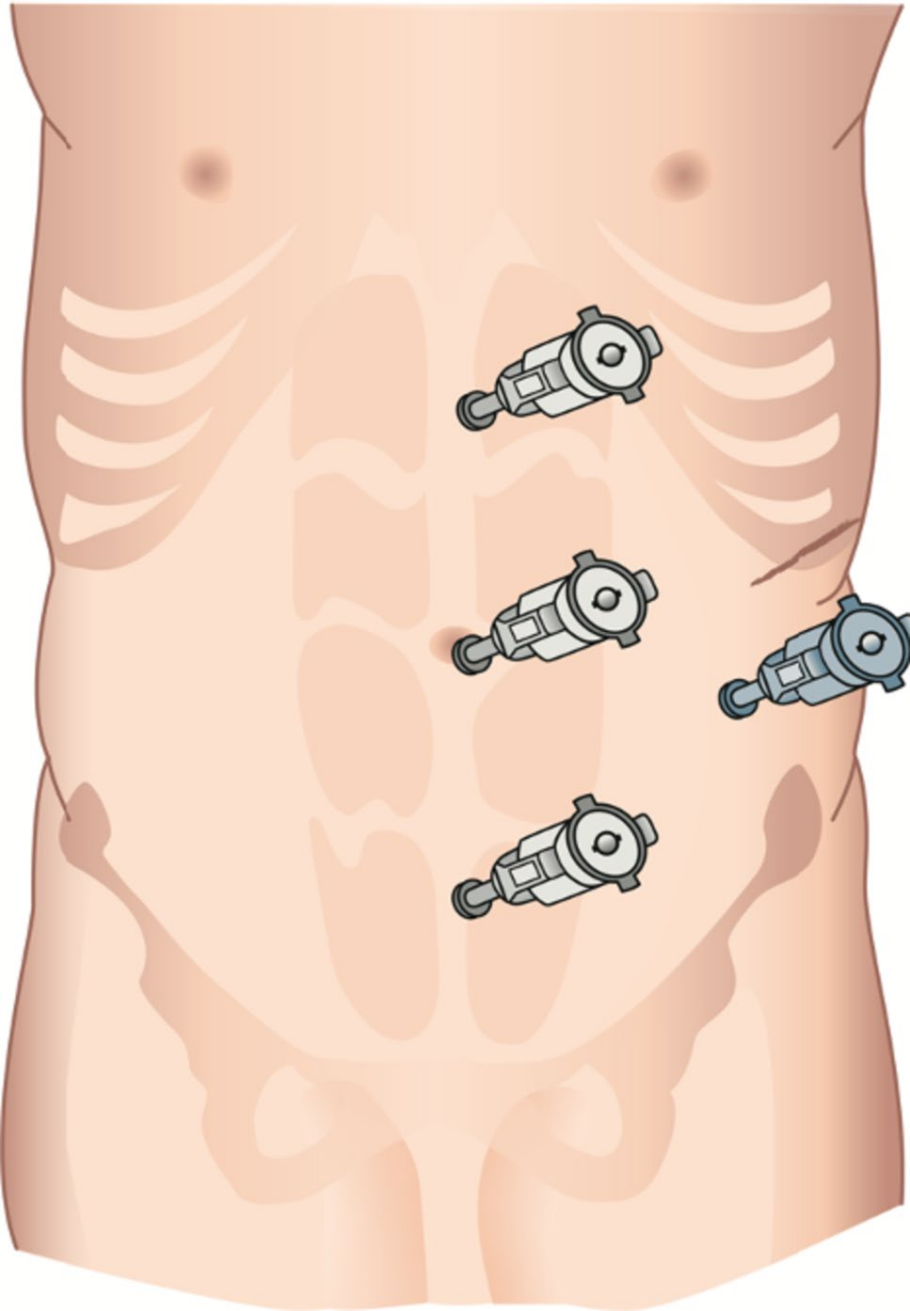
Trocar placement for r-TAPP-procedure (grey: 8-mm trocar, blueish: 12-mm balloon trocar)

R-TAPP was used for lateral, lumbal hernia after open nephrectomy, and the patients were positioned in a lateral position. First, an open introduction of a balloon trocar (Kii® Balloon, Applied Medical, Rancho Santa Margarita, CA, USA) was performed approximately 4-cm medial and caudal to the medial end of the incision. Then three 8-mm trocars were inserted in a caudal to cranial line, approximately 8 cm medially to the incision (Fig. 5), and after docking, preperitoneal preparation and mesh placement were performed similar to the publication of Di Giuseppe and colleagues [19].

In all patients, a light-weighted, large porous, and either non-resorbable (Dynamesh®-CICAT, FEG Textiltechnik, Aachen, Germany; BARD SoftMesh, C. R. Bard GmbH, Karlsruhe, Germany) or partially resorbable meshes (Ultrapro®, Ethicon GmbH, Norderstedt, Germany) were used. All hernia defects and the posterior rectus fascia were closed with slowly resorbable sutures (STRATAFIX® spiral, Ethicon GmbH, Norderstedt, Germany).

#### Open sublay hernia repair

For open sublay hernia repair, the skin incision was made above the hernia, and the hernia sack was mobilized. After repositioning of the hernia, the rectus sheet was opened on both sides, and the posterior layer of the rectus sheet was dissected away from the rectus abdominis muscle. The dissection was extended laterally to the linea semilunaris to facilitate a mesh overlap of at least 5 cm to each site. Next, the posterior layer of the rectus sheet was closed by a running suture (Prolene™ 2–0, Ethicon GmbH, Norderstedt, Germany). The mesh (Ultrapro®, Ethicon GmbH, Norderstedt, Germany) was placed in a retromuscular position and fixed with sutures (Prolene™ 2–0, Ethicon GmbH, Norderstedt, Germany). Following this, wound drainage was positioned above the mesh to the surgeon’s discretion, and the anterior layer of the rectus sheet was closed by a running suture (Prolene™ 2–0, Ethicon GmbH, Norderstedt, Germany).

#### eMILOS hernia repair

The procedure was performed as described by Reinpold and colleagues [20]. Shortly, a skin incision of the size of the hernia but maximum of 4 cm directly over the hernia was made. The hernia sack was mobilized and repositioned into the abdominal cavity. Subsequently, the rectus sheet was entered at both sides, and the rectus abdominal muscle was released from the posterior layer of the rectus sheet. After creating a sufficient space, the posterior layer of the rectus sheet was closed by a running suture (PDS™ Plus 2–0, Ethicon GmbH, Norderstedt, Germany), and the Alexis wound retractor (Alexis Laparoscopic System, Applied Medical, Rancho Santa Margarita, CA, USA) or balloon trocar (Applied Medical, Rancho Santa Margarita, CA, USA) was brought into place. Carbon dioxide was insufflated to a pressure of 14 mmHg. The 12-mm camera trocar (Kii® Fios®, Applied Medical, Rancho Santa Margarita, CA, USA) was inserted via the Alexis and two additional 5-mm trocars (Kii® Fios®, Applied Medical, Rancho Santa Margarita, CA, USA) were placed on both sides at the lateral border of the rectus sheet. The anterior layer of the rectus sheet was closed by a running suture (PDS™ Plus 2–0, Ethicon GmbH, Norderstedt, Germany). In all cases, a partially resorbable mesh (Ultrapro®, Ethicon GmbH, Norderstedt, Germany) was used.

### Data collection

Data for this study was collected retrospectively from medical records and patient charts. Data collection included patient demographics (age, sex, body mass index (BMI), preoperative risk factors (smoking, immunosuppression, connective tissue disorders, previous wound infection), hernia characteristics (localization, size, previous surgical procedure), operating time, dimension of the mesh, and postoperative outcome (LOS, complications, morbidity).

Follow up in the RAHR group was carried out by telephone interviews. With a standardized questionnaire, the number of days after the operation with need for analgetic and ongoing need for analgesia, rate of rehospitalization, wound infections, hernia recurrence, and subjective patient satisfaction (scale 0–100%) were evaluated.

For follow-up in Sublay and eMILOS groups, medical records in our patient database were reviewed.

### Statistical analysis

Statistical analysis was carried out using SPSS (version 29.0.1.0, IBM, New York, USA). Patients in the RAHR group were 1:2 matched by age, sex, BMI, patient risk factors, and hernia size to patients in the Sublay group using propensity score matching (propensity score matching for SPSS version 3.0.4, Cornell University/University of Tuebingen, New York, USA/Tuebingen, Germany) with nearest neighbor method and a caliper of 0.2. After matching, non-unbalanced covariates were present. Propensity score matching to the eMILOS group was not possible because of the smaller number of patients in this group.

Data was tested for normal distribution with the Shapiro–Wilk test. For comparison of normally distributed data, a two-tailed unpaired *t*-test was used. Comparison of non-normally distributed data was performed by the Mann–Whitney *U* test.

## Results

### Patient demographics and risk factors

Twenty-one patients who underwent robotic-assisted incisional hernia repair, 42 with sublay hernia repair, and 19 with eMILOS hernia repair were evaluated in this study. Among the RAHR and the eMILOS groups, there were no conversions to open surgery.

The median (range) age was 57 (40–66) years in the RAHR group, 56 (26–81) years in the Sublay group and 63 (34–81) years in the eMILOS group, without significant differences between the groups. There was no significant difference regarding the sex of the patients. Patients in the RAHR group had a mean (SD) BMI of 29 (± 7.2 SD) kg/m^2^, 28 (± 5.3 SD) kg/m^2^ in the Sublay group and 28 (± 5.9 SD) kg/m^2^ in the eMILOS group (Table 1). The BMI did not differ significantly between the groups. Prevalence of overweight was similar in RAHR (33%) and Sublay group (35%). The proportion of obese patients was equal in both groups (38.1%). In the eMILOS group, 42% of the patients were overweight, and 21% were obese.

**Table 1:** Overall patient demographics and risk factors for incisional hernia

		RAHR	Sublay	eMILOS
*N*		21	42	19
Sex				
	Female	10 (47.6%)	17 (40.5%)	10 (52.6%)
	Male	11 (52.4%)	25 (59.5%)	9 (47.4%)
	Ratio F:M	1:1.10	1:1.47	1:0.90
Age (yrs)		57 (40–66)	56 (26–81)	63 (34–81)
BMI (kg/m^2^)		29.1 ± 7.2	28.5 ± 5.3	28.3 ± 5.9
Risk factors				
	Diabetes	6 (28.6%)	13 (30.9%)	2 (10.5%)
	Medical immunosuppression	7 (33.3%)	15 (35.7%)	4 (21.1%)
	Postoperative wound infection	1 (4.8%)	2 (4.8%)	1 (5.3%)
	Smoking	7 (33.3%)	13 (30.9%)	3 (15.8%)
	Connective tissue disorders	0	0	0

Data are shown as *n* (%), median (range) or mean ± SD

*BMI* body mass index

The most common risk factor for incisional hernia among the groups was immunosuppression, followed by smoking and diabetes. Only one patient in the RAHR and eMILOS groups and two patients in the Sublay group had a wound infection after primary surgery. Connective tissue disorders were not present in the study population (Table 1).

### Hernia size and localization

Median (range) hernia size in the RAHR group was 8 (3–12) cm, 7 (2–16) cm in the Sublay group, and 7 (4–14) cm in the eMILOS group. There were no significant differences in hernia size between the groups.

Among the groups, the most frequent hernia localization (according to the EHS classification) was M2 followed by M3. Lateral hernias (L1-L4) were less frequent in the RAHR and Sublay groups and not present in the eMILOS group (Table 3).

### Procedural data

In 16 of 21 patients (76.2%) in the RAHR group, hernia repair was performed in r-RMHR technique with TAR in 56.3% of the cases. In one patient, r-RMHR was combined with robotic scrotal hernia repair (r-TAPP, patient 12). Two patients (9.5%) were operated with eTEP technique. R-TAPP procedure was conducted in three patients (14.3%), of which lumbal hernia repair was combined with umbilical hernia repair in one case (patient 13) (Table 5). Median (range) operating time was 219 (122–311) min and significantly longer than in the Sublay group (median (range) 109.5 (48–270) min, *p* < 0.001) and in the eMILOS group (median (range) 123 (100–192) min, *p* < 0.001) (Table 3).

The meshes applied in the RAHR group were significantly larger than in the Sublay group (mean (SD) 529 ± 311 cm^2^ vs. 356 ± 231, *p* = 0.037), but there was no difference compared to the eMILOS group (mean (SD) 596 ± 266 cm^2^). Wound drainage was placed in 76.2% of the patients in the RAHR group, 85.7% in the Sublay group, and 100% in the eMILOS group (Table 3).

The highest overall complication rate was seen in the Sublay group (*n* = 5, 12%), followed by the RAHR group (*n* = 2, 10%), and the eMILOS group (*n* = 1, 5%). Of the 5 patients with complications in the Sublay group, there were three patients with seroma, of which two received interventional drainage, and one patient with an abscess requiring operative revision. Another patient showed an incarcerated early recurrent hernia with consecutive ileus on postoperative day 2 and therefore had to be reoperated. In the RAHR group, two seromas occurred (patient 4 and 18) with the need for interventional drainage in one case (patient 4). One patient in the eMILOS group was reoperated because of a seroma inadequately drained interventionally.

The median (range) length of stay in the RAHR group was 3 (2–7) days and was significantly shorter compared to the Sublay group with a median (range) 5 (1–9) days, *p* = 0.032). There was no significant difference in LOS when comparing the cohort to the eMILOS group, with a median (range) 4 (2–12) days LOS (Table 3).

### Follow-up

Median (range) follow-up duration in the RAHR group was 61 (33–132) days with a follow-up rate of 86%. Three patients were lost to follow-up. The median (range) need for painkillers after the operation was 4 (2–14) days. As of last follow-up, there were no cases of ongoing need for painkillers, chronic pain, rehospitalization, or wound infection in this group (Table 5). The median (range) follow-up duration in the Sublay group was 760 (42–1740) days, and 332 (36–840) in the eMILOS group, and was significantly longer in both of these groups compared to the RAHR group (*p* < 0.001).

Three patients in the Sublay group had recurrent hernia, whereas no recurrent hernia was seen in the RAHR and eMILOS groups (Table 3).

### Subgroup analysis of midline hernia

After exclusion of lateral hernia, 16 patients remained in the RAHR group and 32 patients in the Sublay group, respectively. eMILOS group did not include any lateral hernia; therefore, all of the 19 patients were included in the subgroup analysis.

Median age of the patients and gender distribution did not differ from the overall analysis. There were no significant differences in BMI between the groups. The incidence of risk factors was similar to the overall analysis (Table 2).

**Table 2:** Patient demographics and risk factors for midline incisional hernia

		RAHR	Sublay	eMILOS
*N*		16	32	19
Sex				
	Female	9 (56.3%)	15 (46.9%)	10 (52.6%)
	Male	7 (43.7%)	17 (53.1%)	9 (47.4%)
	Ratio F:M	1.0:0.78	1:1.13	1:0.90
Age (yrs)		57 (40–65)	56 (26–81)	63 (34–81)
BMI (kg/m^2^)		27.1 ± 5.6	28.5 ± 5.5	28.3 ± 5.9
Risk factors				
	Diabetes	3 (18.8%)	7 (21.7%)	2 (10.5%)
	Medical immunosuppression	6 (37.5%)	11 (34.4%)	4 (21.1%)
	Postoperative wound infection	1 (6.3%)	2 (6.3%)	1 (5.3%)
	Smoking	6 (37.5%)	8 (25.0%)	3 (15.8%)
	Connective tissue disorders	0	0	0

Data are shown as *n* (%), median (range) or mean ± SD

*BMI* body mass index

Median (range) hernia size in the RAHR group was 7.5 (3–12) cm, 7.5 (2–16) in the Sublay group, and 7 (4–14) cm in the eMILOS group. There were no significant differences in hernia size between the groups (Table 4).

**Table 3:** Overall hernia characteristics and procedural data

		RAHR	Sublay	eMILOS
*N*		21	42	19
Hernia size (cm)		8 (3–12)	7 (2–16)	7 (4–14)
EHS classification	M1	0	3 (7%)	0
	M2	11 (52%)	15 (36%)	9 (47%)
	M3	4 (19%)	11 (26%)	8 (42%)
	M4	1 (5%)	2 (5%)	2 (11%)
	M5	0	1 (2%)	0
	L1	1 (5%)	3 (7%)	0
	L2	1 (5%)	2 (5%)	0
	L3	0	2 (5%)	0
	L4	3 (14%)	3 (7%)	0
Multiple hernia		10 (48%)	21 (50%)	9 (47%)
Operating time (min)		219 (122–311)	109.5 (48–270)^#^	123 (100–192)^#^
Mesh size (cm^2^)		529 (± 311)	356 (± 231)^#^	596 (± 266)
Wound drainage		16 (76%)	36 (85.7%)	19 (100%)
Overall complications		2 (10%)	5 (12%)	1 (5%)
Complications according to Clavien-Dindo classification	Grade I	1 (5%)	1 (2.4%)	0
	Grade II	0	0	0
	Grade IIIa	1 (5%)	2 (4.8%)	0
	Grade IIIb	0	2 (4.8%)	1 (5%)
	Grade IV	0	0	0
	Grade V	0	0	0
Length of stay (*d*)		3 (2–7)	5 (1–9)^#^	4 (2–12)
Follow-up				
	Duration (*d*)	61 (33–132)	760 (42–1740)^#^	332 (36–840)^#^
	Lost to follow-up	3 (14%)	5 (11%)	4 (21%)
	Hernia recurrence	0 (0%)	3 (9%)	0 (0%)

Data are shown as *n* (%) or median (range) or mean ± SD

^#^Significant vs. RAHR (*p* < 0.05)

As in the overall analysis, operating time was significantly higher in RAHR group (216.5 (122–311) min) compared to sublay (112.5 (48–270) min, *p* < 0.001) and eMilos group (median (range) 123 (100–192) min, *p* < 0.001).

The meshes applied in the RAHR group were significantly larger than in the sublay (mean (SD) 596 ± 321 cm^2^ vs. 385 ± 220, *p* = 0.035). Compared to the eMILOS group (mean (SD) 596 ± 266 cm^2^), there was no significant difference in mesh size. Wound drainage was placed in 76.2% of the patients in the RAHR group, 85.7% in the Sublay group, and 100% in the eMILOS group (Table 3). Similar to the overall analysis, the highest rate of complications was seen in the Sublay group (*n* = 4, 12%), followed by the RAHR group (*n* = 1, 6%), and the eMILOS group (*n* = 1, 5%). Three patients with complications in the Sublay group had seroma, of which two received interventional drainage. Another patient had an abscess requiring operative revision. In the RAHR, one seroma occurred (patient 18) which was treated without drainage. One patient in the eMILOS group was reoperated because of a seroma inadequately drained interventionally.

Similar to the overall analysis, the median (range) length of stay in the RAHR group (3 (2–7) days) was significantly shorter compared to the Sublay group (5 (1–9) days, *p* = 0.03), but not significantly different compared to the eMILOS group. Recurrence rate in the Sublay group was 6% (*n* = 2); whereas, there were no recurrent hernia in RAHR and eMILOS groups. The follow-up duration in the Sublay group and eMILOS group was significantly longer compared to RAHR group (*p* < 0.001) (Table 4).

**Table 4:** Characteristics and procedural data of midline incisional hernia

		RAHR	Sublay	eMILOS
*N*		16	32	19
Hernia size (cm)		7.5 (3–12)	7.5 (2–16)	7 (4–14)
EHS classification	M1	0	3 (9%)	0
	M2	11 (69%)	15 (47%)	9 (47%)
	M3	4 (25%)	11 (35%)	8 (42%)
	M4	1 (6%)	2 (6%)	2 (11%)
	M5	0	1 (3%)	0
Multiple hernia		9 (56%)	18 (56%)	9 (47%)
Operating time (min)		216.5 (122–311)	112.5 (48–270)^#^	123 (100–192)^#^
Mesh size (cm^2^)		596 (± 321)	385 (± 220)^#^	596 (± 266)
Wound drainage		12 (75%)	29 (90.6%)	19 (100%)
Overall complications		1 (6%)	4 (12%)	1 (5%)
Complications according to Clavien-Dindo classification	Grade I	0	1 (3%)	0
	Grade II	0	0	0
	Grade IIIa	1 (6%)	2 (6%)	0
	Grade IIIb	0	1 (3%)	1 (5%)
	Grade IV	0	0	0
	Grade V	0	0	0
Length of stay (*d*)		3 (2–7)	5 (1–9)^#^	4 (2–12)
Follow-up				
	Duration (*d*)	78 (35–132)	728 (42–1740)^#^	332 (36–840)^#^
	Lost to follow-up	2 (12.5%)	5 (15.6%)	4 (21%)
	Hernia recurrence	0 (0%)	2 (6%)	0 (0%)

Data are shown as *n* (%) or median (range) or mean ± SD

^#^Significant vs. RAHR (*p* < 0.05)

## Discussion

Robotic hernia surgery is a safe and well-tolerated technique for the repair of incisional hernia. Our experience with robotic-assisted repair is in line with previously published case series for RAHR for complex ventral and lateral hernia [14, 21–24]. We demonstrated shorter length of hospital stay compared to the open sublay technique, no intra-operative complications or conversions and no postoperative bleeding. Patient satisfaction was extremely high at 100%, and the need for painkillers was low with a median of 4 days. In the short-term follow-up period, no recurrences were observed or reported and was lower than in the Sublay group and comparable to the eMILOS group. In our study cohort, a large proportion of patients had complex incisional hernia, and 7 patients were under immunosuppressive medication, with five under immunosuppression due to liver transplantation. Despite this, postoperative morbidity and rates of surgical complications were low. No SSIs were observed, and only two patients had asymptomatic postoperative seroma. The complication rate of 9.6% was lower than in the Sublay group (17%) but higher than in the eMILOS group. However, significantly fewer patients in the eMILOS group were smokers and immunosuppressed, which may contribute to the lower complication rate in this group than in the RAHR cohort. Because no patient in the eMILOS group had a lateral hernia, we performed a subgroup analysis of patients with midline hernia only. This subgroup analysis revealed very similar results compared to the overall analysis.

**Table 5:** Surgical procedure, operating time, and hernia characteristics in the RAHR group

No	Surgical procedure	Site of hernia	Size of hernia (cm)	Previous surgical procedure	Operating time (min)	Mesh size (cm)
1	eTEP	Midline incision	3	Tumor debulking	213	22 × 18
2	r-RMHR	Midline incision	3	Ileostoma reversal, left hemicolectomy	191	15 × 10
3	r-RMHR	Epigastric incision (trocar)	3	DaVinci prostatectomy	138	15 × 10
4	r-RMHR	Reversed L-shaped incision	5	Right hemihepatectomy	294	30 × 20
5	r-RMHR	Midline incision	12	Aortic aneurym repair	220	45 × 30
6	r-RMHR + TAR	Reversed L-shaped incision	10	Liver transplantation	221	30 × 30
7	r-RMHR + TAR	Midline incision	12	Adhesive small bowel obstruction	237	30 × 15
8	r-RMHR + TAR	Reversed L-shaped incision	10	Liver transplantation	311	30 × 30
9	r-RMHR + TAR	Reversed L-shaped incision	10	Liver transplantation	274	22 × 19
10	r-RMHR + TAR	Reversed L-shaped incision	12	Liver transplantation	264	25 × 22
11	r-RMHR + TAR	Inverted T-shaped incision	8	Open hiatal hernia repair	173	25 × 20
12	r-RMHR + r-TAPP	Reversed L-shaped incision	10	Liver transplantation	291	22 × 15
13	r-TAPP	Lumbar incision	6	Right-sided nephrectomy	238	20 × 15
14	r-RMHR + TAR	Reversed L-shaped incision	6	Resection liver segment VII/VIII	164	20 × 20
15	r-RMHR + TAR	Inverted T-shaped incision	10	Distal pancreaticosplenectomy	227	30 × 30
16	r-TAPP	Lumbar incision	7	Left-sided nephrectomy	190	17 × 15
17	eTEP	Midline incision	3	Explorative laparotomy, adhesiolysis	122	30 × 20
18	r-TAPP	Lumbar incision	10	Partial left-sided renal resection	208	30 × 15
19	r-RMHR	Midline incision	8	Low anterior resection	219	20 × 15
20	r-RMHR + TAR	Midline incision	7	Explorative laparotomy, adhesiolysis	137	30 × 20
21	r-RMHR	Inverted T-shaped incision	7	Total gastrectomy	156	30 × 30

*r-RMHR* robotic retro-muscular hernia repair, *TAR* transversus abdominis release, *eTEP* enhanced-view totally extraperitoneal hernia repair, *r-TAPP* robotic-assisted transabdominal preperitoneal hernia repair

The existing studies describe three robotic mesh placement techniques: the robotic IPOM, trans-abdominal preperitoneal placement (rTAPP), and retro-muscular techniques, either trans-abdominal or without entering the abdominal cavity (TARUP/rRS and robotic eTEP) [25]. In comparison to rTAPP, the robotic IPOM seems to be associated with a higher frequency of postoperative complications, higher morbidity, and a lower mesh-to-defect ratio [26, 27]. Robotic eTEP also showed a lower complications rate and a higher mesh-to-defect ratio compared to robotic IPOM suggesting an extraperitoneal mesh placement should be preferred [28]. However, a recent published randomized-controlled trail did not reveal a significant difference between robotic eTEP and robotic IPOM regarding postoperative pain and procedural costs [29].

As shown in our series, for each hernia, an individually tailored surgical approach can be used, depending on localization, size, intraabdominal adhesions and patients’ morbidity. This includes not only ventral hernia of the midline and transverse laparotomies lateral of the rectus sheet but even lumbal and intercostal hernia after nephrectomy that has recently been described in some case series and a register analysis [19, 24, 30, 31].

The robotic-assisted surgical devices with wristed and articulated instruments allow a higher degree of freedom of movement, making intracorporal suturing easy, and the preparation of a large preperitoneal retro-muscular room for mesh placement is also far easier (and faster) to perform [22]. Control of the scope by the surgeon as well as 3D visualizations provide a more stable and reproducible image. Moreover, the seated positioning of robotic surgery improves ergonomics which is an important factor in times of increasing staff costs and decreasing number of surgeons [32, 33]. Together, these factors have the potential to overcome at least some of the obstacles which surgeons face with the laparoscopic approaches.

In general, hernia surgery should be embedded in a patient pre-habilitation program including weight reduction, smoking cessation, and drug adaption to optimize the postoperative short-term and long-term results [34]. Additionally, hernia-specific pre-treatments, such as preoperative botulinum toxin injection and progressive pneumo-peritoneum and traction systems during surgery, improve the chance of midline closure which is essential for the patient outcome [35–37]. However, some robotic hernia surgeons have postulated that the intra-operative relaxation and capno-peritoneum during the preparation phase of the procedure lead to a significant stretching of the abdominal wall allowing a closure in virtually every case, especially when combined with a TAR. In our series, even in large hernias of 10 cm or more, a midline closure was possible without a pre-treatment.

Robotic hernia surgery is critically debated in the surgical society: the main criticism of RAHR is the higher procedure-related costs due to longer operating times as well as the higher purchasing, material, and operating costs against the background of its unclear clinical benefits [17, 23, 38]. For inguinal, umbilical, and smaller ventral hernia, the financial disadvantage cannot be questioned; but in case of complex hernia, the potential clinical benefits might outweigh the higher procedure-related costs. Accordingly, Dauser et al. calculated that the higher procedure-related costs of robotic-assisted techniques were balanced by a shorter hospital stay and a lower 30-days readmission rate compared to open hernia surgery [38]. Baur and colleagues described a reduction of case costs compared to the IPOM technique basically due to cheaper meshes [39]. This study also shows a significant longer operating time for RAHR in comparison to open sublay and eMILOS techniques. In line with Dauser et al., LOS in the RAHR group was shorter than in the Sublay group, but there was no difference compared to the eMILOS group.

In our study, there were no differences seen between the RAHR and the eMILOS group with regard to LOS, mesh size, and recurrence rate. However, the significantly larger meshes applied in the RAHR group compared to the Sublay group may indicate a simplified preparation of the retro-rectus space by the robotic and endoscopic technique. The resulting larger overlapping area of the mesh may contribute to the lower recurrence rates compared to the open sublay technique. In view of the above and against the background of the partly complex cases of this series, this may indicate that due to the advantages of the robotic technique, less experienced surgeons may be able to perform demanding procedures, which can otherwise only be performed laparoscopically by surgeons with vast laparoscopic experience. Therefore, the more complex the hernia and the need for component separation are, the more beneficial the use of the robotic platform may become.

In health care systems such as the German diagnosis-related groups (DRG) system, robotic surgery does not lead to a higher reimbursement compared to standard minimally invasive techniques. This puts healthcare providers under economic pressure and may result in a limited use of robotic-assisted techniques if the clinical benefits cannot be shown. Vice versa, an implementation of robotic-assisted incisional hernia procedures within the DRG system may lead to a wider implementation of this technique and may help to reduce the economic burden of incisional hernia in the long term.

The study has several limitations: it provides only a small number of cases in the study group as well as in both control groups with an even lower number in the subgroup analysis of midline hernia. Because of the retrospective analysis, without a structured follow-up comparable data of clinical outcome in terms of the need for painkillers, chronic pain, rehospitalization, and wound infection is lacking for the Sublay and eMILOS groups. Furthermore, this study presents the clinical results during the learning curve of the robotic hernia surgery program. With this in mind, the short-term outcome appears to be good and is comparable to outcomes described in the current literature; however, the long-term outcomes are not yet known.

## Conclusion

In summary, robotic incisional hernia repair promises to combine the best of both worlds: the advantages of open hernia repair with low recurrence rates and the ability to perform complex procedure (such as transverse abdominis release) and of laparoscopic surgery with its low rates of SSIs and less pain. The higher procedure related costs may be outweighed by the low complication and recurrence rates in complex abdominal wall reconstructions. However, a clinical benefit compared to minimal invasive sublay techniques is not yet proven and needs to be further investigated in prospective randomized trials.
